# Corticotropin‐releasing factor is involved in acute stress‐induced analgesia and antipruritus

**DOI:** 10.1002/brb3.2783

**Published:** 2022-10-09

**Authors:** Xiao‐Dong Wang, Hao‐Miao Bai, Xiao‐Lan Li, Lin‐Fang Zhang, Fei Li, Yang Bai, Zhen‐Yu Wu, Shang‐Qing Liu, Hui Li

**Affiliations:** ^1^ Department of Human Anatomy, Histology and Embryology and K.K. Leung Brain Research Centre Air Force Military Medical University Xi'an China; ^2^ Department of Emergency Medicine Inner Mongolia Armed Police Corps Hospital Hohhot China; ^3^ Department of Human Anatomy, School of Basic Medical Sciences and Forensic Medicine North Sichuan Medical College Nanchong China; ^4^ Department of Anatomy Medical School of Yan'an University Yan'an China; ^5^ Department of Neurosurgery General Hospital of Northern Theater Command Shenyang China

**Keywords:** analgesia, antipruritus, CRF, itch, pain, stress

## Abstract

**Background:**

Under the condition of stress, the hypothalamic–pituitary–adrenal axis (HPA axis) is activated and causes the secretion of corticotropin‐releasing factor (CRF). Previous studies have demonstrated that CRF is involved in the regulation of pain and itch. Thus, it remains worthy to explore whether the desensitization of pain and itch under high‐intensity acute stress (such as high fear and tension) is related to the sharp increase of CRF.

**Methods:**

Forced swimming was used to simulate acute stress. ELISA and pharmacological methods were conducted to observe the effects of forced swimming on acute pain or itch and the relationship between blood CRF content and itch or pain behavior. Intracerebroventricular (ICV) administration of CRF was conducted to examine the effects of CRF on acute pain or itch. Intrathecal administration of CRF receptor agonist or antagonist was conducted to examine the receptor mechanisms of the regulatory role of CRF in pain and itch.

**Results:**

ELISA experiment showed that the serum CRF in mice reached its peak within 5–10 min after acute stress (forced swimming). Behavioral data showed that the scratching behavior induced by itch agents decreased after acute swimming, while the mechanical pain threshold increased significantly. The inhibitory effect of acute stress on pain and itch is mediated by CRF receptor2 (CRFR2). Then, ICV injection of CRF was used to simulate the massive release of CRF under acute stress, and we observed that the scratching behavior induced by histamine or chloroquine was significantly inhibited after ICV injection of CRF. The above effects of CRF are mainly mediated by CRFR2. These results suggest that 5–10 min after acute stress, a large amount of CRF is released into the blood from the hypothalamus, which significantly inhibits acute pain and itch by acting on CRFR2. ICV injection of CRF can replicate the antipruritus effects of acute stress.

**Conclusions:**

The present study investigated the mechanism of acute stress‐induced analgesia and antipruritus and provided theoretical support for the treatment of pain and itch.

## Graphical Abstract

In this study, we found that the sharp increase of CRF secretion under acute stress led to the desensitization of pain and itch sensation, namely, stress analgesia and stress antipruritus. This effect was mediated by CRF2 receptor.

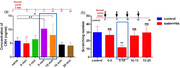

## 1 INTRODUCTION

Corticotropin‐releasing factor (CRF) is a 41 peptide secreted by the parvocellular division of hypothalamic paraventricular nucleus (PVN). As the central driving force of HPA axis excitation, CRF plays a key role in neuroendocrine regulation during a stress response. Recent studies have shown that CRF, a widely distributed neurotransmitter/modulator in the central nervous system, is involved in many important physiological functions, such as visceral sensation, cardiovascular activity, emotional response, learning, memory, and so forth. The effect of CRF is achieved by binding to the CRF receptor. Mammalian CRF receptors (CRFR) are mainly divided into two types: CRFR1 and CRFR2, both of which are Gs‐type G protein‐coupled receptors and widely distributed in the central nervous system. However, their distribution regions are different, implying that they may mediate different physiological functions (Fu & Neugebauer, 2008; Ma et al., 2020).

Stress refers to the nonspecific response of the body caused by excessive or harmful stimuli that destroy the homeostasis of the internal environment of the body, which can be divided into acute stress and chronic stress. Moderate stress plays an important role in maintaining the normal physiological state of the body, while excessive or persistent stress will lead to a series of pathological phenomena. As a key regulatory factor in stress response, CRF produces a series of biological effects through its receptor, mobilizes various systems of the body to respond to stress stimuli, and regulates endocrine, autonomic, immune, and behavioral responses (Bakshi & Kalin, 2000). Some studies have found that an ultra‐short positive feedback mechanism may contribute to the rapid increase in the secretion of CRF in the PVN during stress, which means that CRF acts on CRFR1 in the PVN in an autocrine manner, thereby promoting autocrine secretion (Jezova et al., 1999). CRFR2 is necessary for the recovery of a stress response. CRFR2‐deficient animals are highly sensitive to stress. After restraint stress, plasma adrenocorticotropic hormone (ACTH) and corticosterone levels peaked more rapidly than the control group and were maintained for a longer time (Coste et al., 2000). During acute stress, a large amount of CRF is released, which mediates in acute stress response, while chronic stress will cause sustained a release of CRF in medium and low doses, which can cause a series of chronic stress responses including anxiety and depression (Borges et al., 2013).

Acute stress produces antinociceptive effects that is also called stress‐induced analgesia (SIA). For example, the exposure of mice already under painful stimuli to cats significantly reduced their pain response (Butler & Finn, 2009). In addition, there are two types of forced swimming‐induced SIA: under the stress state of weak stimulation intensity (warm water and short stimulation time), the production of SIA is related to the activation of opioid peptides, which can be stimulated by the µ‐opioid receptor blocker naloxone (Parikh et al., 2011). Another SIA is produced under strong stimulation intensity (cold water and long stimulation time), and the production of this analgesic effect is independent of opioid peptides, which cannot be blocked by naloxone but can be blocked by neurotensin (NT) (Mogil et al., 1996). Similarly, Spradley et al. (2012) observed that swimming in cold water inhibited the scratching behavior induced by cheek injection of 5‐HT in rats. However, under the condition of acute stress (especially fear and anxiety), both pain and itch sensation become dull or disappear completely, and whether it is related to the increase of CRF secretion in this state has not been reported. This study mainly simulated the acute stress state by forced swimming and detected the changes of CRF content in the blood of mice at different time points after forced swimming and corresponding changes in acute pain and acute itch behavior.

## 2 MATERIAL AND METHODS

### 2.1 Experimental animals

Adult male C57/BL6 mice (10–12 weeks) weighing 20–25 g (provided by the Experimental Animal Center of Air Force Military Medical University) were selected. According to breeding and experimental operating procedures of our laboratory, the room temperature is maintained at around 22–25°C with appropriate air humidity and ventilation, and the mice can freely get access to food and water. All operating methods of this experiment followed the rules of the Air Force Military Medical University Ethics Committee on Animal Use and Laboratory Animal Management. The number of animals used in surgery and behavioral experiments was minimized and the trauma to animals was reduced.

### 2.2 Experimental design


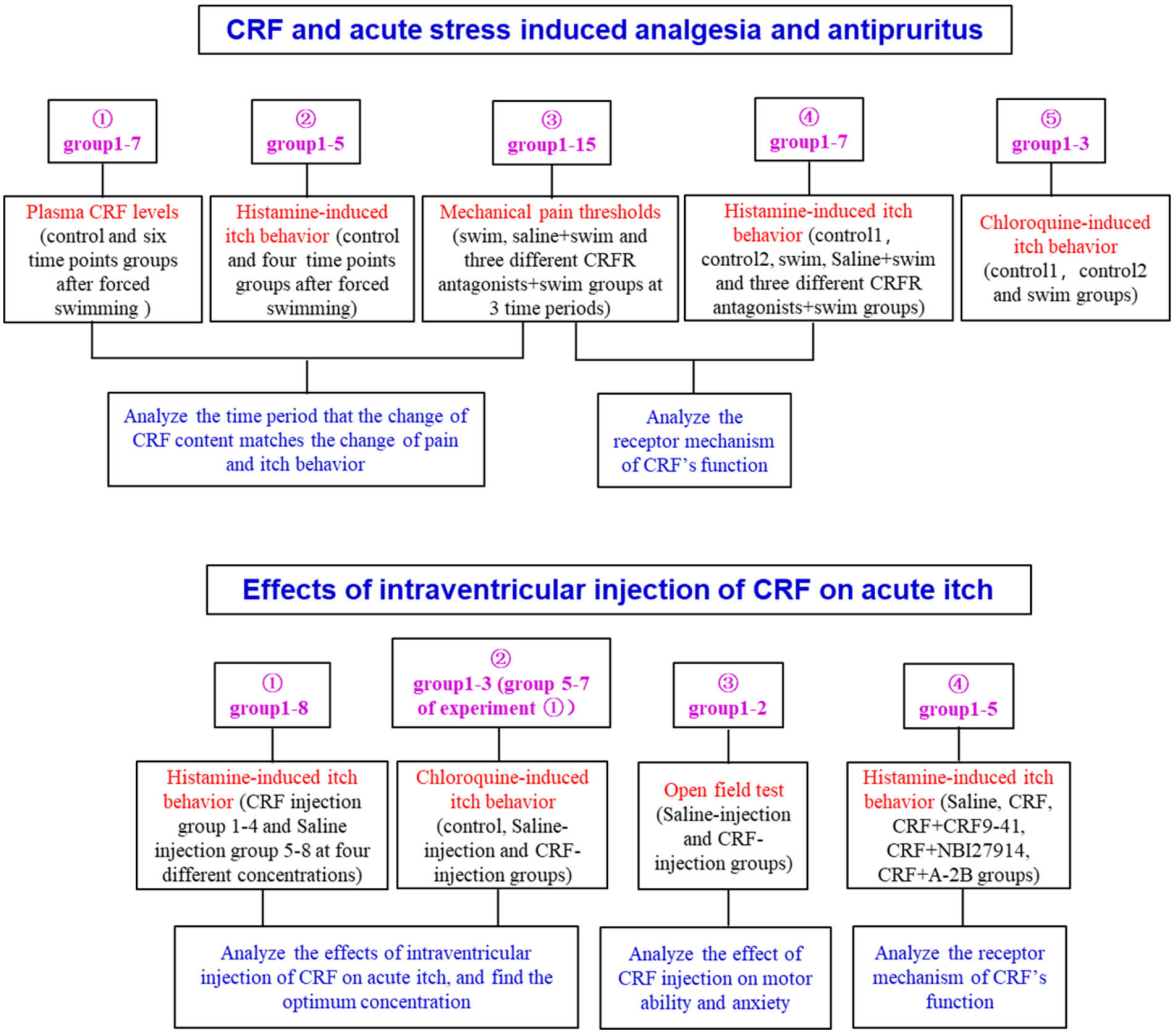


The visual representation of the experimental design.

The experimental design includes two parts. The first part (upper figure) is the design of “CRF and acute stress induced analgesia and antipruritus”, and this part includes five different experiments. The upper five text boxes represent the five experiments and the group number. The middle five text boxes represent the experimental content and grouping. The bottom two text boxes show the experimental objective. The second part (lower figure) is the design of “Effects of intraventricular injection of CRF on acute itch”. The three rows of text boxes represent the same meaning as the first part. The mice of group 1‐3 in the second experiment were the same mice in group 5‐7 in the first experiment.

### 2.3 Relationship between the content of CRF in arterial blood and histamine‐induced scratching times in mice at different time points after acute stress

#### 2.3.1 ELISA detection of CRF content in arterial blood at different time points after acute stress

Forty‐two C57 mice were randomly divided into seven groups with six mice in each group and denoted as A1–A7 groups. The mice in group A1 were reared in an environment that avoided stress as much as possible. After anesthetized, the eyeballs of the mice were directly removed, 1 ml of arterial blood was collected, and then they were sacrificed; mice in group A2 were reared under the same conditions and underwent forced swimming for 3 min (water temperature maintained at about 24°C), and then blood was collected immediately as described before; the mice in the A3–A7 groups underwent forced swimming for 3 min, and then were returned to their original cages, and the mice in the A3–A7 groups were sacrificed for blood sampling at the 3, 5, 10, 15, and 20 min later, respectively. After being kept at room temperature for 1 h, the samples were centrifuged at 3000 rpm for 10 min at 4°C, and then 200 µl of the upper serum was used to perform ELISA tests. The absorbance value was used to determine the protein content. Then, the histogram of CRF content was drawn according to the arterial blood samples at different time points after acute stress.

#### 2.3.2 Detection of histamine‐induced scratching behavior at different time points after acute stress

Thirty C57 mice were selected and divided into five groups. Group 1 was the control group. The mice were injected with 20 µl of histamine at a concentration of 10 mg/ml intradermally within the neck, and then were videotaped for 5 min, and the number of scratches was counted. Groups 2–5 were forced swimming + histamine injection group (swim + His group). In this group, the mice underwent forced swimming for 3 min in appropriate water temperature, respectively. At 0, 5, 10, and 15 min after forced swimming, 20 µl of histamine (the same concentration as before) was intradermally injected into the neck. Then, the mice were videotaped for 5 min, with the number of scratches in each group counted.

### 2.4 The change of mechanical pain threshold in mice at different time periods after acute stress and effects of intrathecal injection of CRFR antagonists on pain threshold

#### 2.4.1 Measurement of mechanical pain threshold

According to our previous study, the threshold of mechanical pain was measured (Yin et al., 2016). To ensure the stability of the experimental results, the animals were acclimated for 1 week before the experiment. We used a double‐blind method for testing, and the experimenter did not know the grouping of animals. The mice were placed in a transparent glass box with a metal mesh (7 cm × 7 cm × 10 cm), and the metal mesh was put about 30 cm away from the experimental table. The mice were allowed to adapt for 30 min before testing. When mice are relatively quiet in the box, von Frey filaments of different force including 0.04 g (2.44), 0.07 g (2.83), 0.16 g (3.22), 0.4 g (3.61), 0.6 g (3.84), 1.0 g (4.08), 1.4 g (4.17) (Shenzhen Reward Co., Ltd) were used to stimulate the plantar skin from bottom to top perpendicular to the center of the hind foot of the mouse each time for 3 s until the fiber filaments are bent in an “S” shape. A positive response was recorded if foot withdrawal, foot addition, or foot lift related to von Frey stimulation occurred, and the time interval between two tests of the same plantar stimulation was 10 min. Only the right hind paw was examined for each mouse. Each stimulus intensity was given five times, and the stimulus intensity that elicited more than three times positive responses were recognized as the paw withdraw threshold (PWT), that is, the mechanical pain threshold.

#### 2.4.2 Intrathecal injection of CRFR antagonists

Animals were acclimated to experimental environment 1 week before the experiment. Then, the mice were treated with skin preparation and sterilization. Then, a syringe with 2 µl of CRFR non‐selective antagonist CRF9‐41 (0.1 nmol/µl) was used for intrathecal injection at the midpoint between the line connecting the anterior superior iliac spine and the spine. When the mouse showed a significant tail swing, the injection direction was transferred from vertical injection to injection at an inclination of 45 degrees.

#### 2.4.3 Experiment grouping and process

Ninety male C57 mice were used in the experiment, and they were divided into 15 groups. The mice in groups 1, 6, and 11 underwent forced swimming (named swim group). Then, mechanical pain thresholds within 0–5, 5–10, and 10–15 min after forced swimming for 3 min were recorded. Finally, the difference in mechanical pain threshold between groups was compared and analyzed. The mice in groups 2, 7, and 12 were slowly injected with 2 µl of normal saline intrathecally, recovered for 2 min, and then underwent forced swimming for 3 min (named sal + swim group). Finally, the mechanical pain thresholds of mice in each group were recorded within 0–5, 5–10, and 10–15 min after forced swimming. The mice in groups 3–4, 8–10, and 13–15 were separately slowly injected with 2 µl of CRF9‐41, NBI27914, and A‐2B intrathecally, recovery for 2 min, and then underwent forced swimming for 3 min (named CRF9‐41 + swim group, NBI27914 + swim group, A‐2B + swim group). Finally, the mechanical pain thresholds of mice in each group were recorded within 0–5, 5–10, and 10–15 min after forced swimming.

### 2.5 Effects of acute stress on acute itch induced by histamine or chloroquine

#### 2.5.1 Effects of acute stress on histamine‐induced itch

Forty‐two male C57 mice were divided into seven groups: Con1 group, Con2 group, swimming group, Sal + swim group, CRF9‐41 + swim group, NBI27914 + swim group, and A‐2B + swim group. In the Con1 group, naive mice were injected with 20 µl (10 µg/µl) histamine intradermally. In the Con2 group, the mice were sprayed with warm water to simulate wet hair conditions and then intradermally injected with histamine. In the swimming group, the mice underwent forced swimming for 3 min and then received intradermal histamine injection in the nape. Mice in the Sal + swim group were intrathecally injected with saline for 2 min, then underwent forced swimming for 3 min, and then were intradermally injected with histamine. Mice in CRF9‐41 + swim group, NBI27914 + swim group, and A‐2B + swim group were separately intrathecally injected with CRF9‐41, NBI27914, and A‐2B for 2 min, then underwent forced swimming for 3 min, followed by intradermal injection of histamine. Video recording was performed for 10 min after histamine injection, and the number of scratches was recorded. The number of scratches between groups was compared, and the effects of CRFR antagonists on histamine itch after acute stress were analyzed.

#### 2.5.2 Effects of acute stress on chloroquine‐induced itch

A total of 18 male C57 mice were divided into three groups, that is, Con1, Con2 and swimming groups respectively. In the Con1 group, normal mice were injected with 20 µl (10 µg/µl) of chloroquine. In the Con2 group, the mice were sprayed with water to simulate a wet hair state and then intradermally injected with chloroquine. In the swimming group, the mice underwent forced swimming for 3 min after intradermal chloroquine injection. The number of scratches for 10 min after the injection of chloroquine was counted. The number of scratches between groups was compared, and the effects of acute stress on chloroquine induced itch were analyzed.

### 2.6 Effects of intraventricular injection of CRF on acute itch induced by histamine or chloroquine

#### 2.6.1 Ventricular catheterization

The mice were anesthetized with 7% chloral hydrate (4 mg/kg) by intraperitoneal anesthesia. After skin preparation, the mice were fixed firmly on the stereotaxic injector. After adequate disinfection, the scalp was incised and opened to both sides to fully expose the Bregma and Lamda points. The injection coordinates were determined according to the mouse brain atlas. Then, the skull was drilled with a dental drill. A trocar with a diameter of 0.3 mm was used for stereotaxic positioning into one ventricle (coordinates: 1 mm posterior to Bregma, 0.35 mm to the right, 2.75 mm deep). Then clear and colorless cerebrospinal fluid could be drained out, suggesting right positioning in the ventricular system. The trocar was closed, and the changes in the vital signs of the mice, especially the respiratory rate, were monitored at the same time. After confirming that the mouse was normal, the trocar was fixed with 1454 glue and dental cement. Then, the animals were returned to the cage to separate feeding after they were fully awake. During the entire operation, the eyes of the mice were smeared with erythromycin ophthalmic ointment, and at the same time, a small piece of paper was used to block the direct light to prevent blindness. After the mice were anesthetized, attention should be paid to maintain normal body temperature.

#### 2.6.2 Effects of intraventricular injection of CRF on histamine‐induced itch

After intracerebroventricular catheterization, the mice were left to recover for 1 week and then underwent intraventricular injection experiment. The 48 recovered mice were divided into two groups equally: CRF injection group (CRF + His) and saline control group (Sal + His). The mice in each group were further equally divided into four groups with CRF injections at different concentrations and four groups with saline injections at different concentrations. After ether‐induced anesthesia, the mice in the CRF group were injected with 0.1 µl, 0.5 µl, 1, and 2 µl of CRF (0.1 nmol/µl) into the ventricle with a microsyringe, respectively. The syringe was removed 1 min later for complete drug injection into the ventricle and then the cannula was closed. After the mice fully recovered their exercise capacity, 20 µl of histamine (10 µg/µl) was intradermally injected into the nape, videotaped for 30 min, and the number of scratches in each group was counted. The treatment of the mice in the normal saline control group were the same those in the CRF group, which received 0.1 µl, 0.5 µl, 1 µl, and 2 µl of normal saline injection into the ventricle, respectively. After the mice completely recovered their exercise capacity, histamine was intradermally injected and scratching number was recorded.

#### 2.6.3 Effects of intraventricular injection of CRF on chloroquine‐induced itch

Eighteen mice with ventricular catheterization that did not receive CRF administration in the part 2.6.2 were equally divided into three groups: the Sal + CQ group, the CRF+CQ group, and the CQ group. The treatment in the former two groups was the same as that describe in the part 2.6.2, with 2 µl saline or CRF (0.1 nmol/µl) injected into the ventricle, respectively. After recovery, the mice were injected with 20 µl of chloroquine (10 µg/µl) intradermally in the nape video. The number of scratches in each group was counted for 30 min. The mice in Group 3 were treated with CQ injection only.

#### 2.6.4 Effects of intraventricular injection of CRF on exploratory behavior in open field test

Fourteen male C57 mice were equally divided into CRF group and saline group. The ventricular catheterization operation was the same as that described before. The mice in the CRF group were given an intraventricular injection of 2 µl CRF (0.1 nmol/µl), while those in the saline group were given an intraventricular injection of 2 µl saline. Then, an open field test was performed on the mice and the movement trajectory were recorded. The differences in total distance and residing time in the central area in each group were analyzed.

#### 2.6.5 Effects of intraventricular injection of CRF receptor antagonists on histamine‐induced itch

Thirty mice were divided into five groups: Sal + His group, CRF + His group, CRF + CRF9‐41+His group, CRF + NBI27914+His group, CRF + A‐2B+His group. The mice in the Sal + His group received intraventricular injection of 2 µl normal saline (0.85%). The mice in the CRF + His group were intraventricularly injected with 2 µl CRF (0.1 nmol/µl). The mice in the other groups were intraventricularly injected with 2 µl CRF9‐41, NBI27914, and A‐2B (0.1 nmol/µl), respectively. Five minutes later, the mice were treated with 2 µl CRF (0.1 nmol/µl). For all the mice, 5 min after CRF injection, they received intradermal injection of 20 histamine (10 µg/µl), and the scratching behavior were recorded. The difference in scratching number among the groups was compared and analyzed.

### 2.7 Statistical analysis

IBM SPSS 13.0 and GraphPad Prism7.0 software packages were used to analyze and plot the results. Before applying any statistics, the normal distribution of the data were first checked. The samples conforming to normal distribution were statistically analyzed using Student's *t*‐test. The data were presented as the mean ± standard error of the mean (SEM). A *p*‐value < .05 was considered statistically significant.

## 3 RESULTS

### 3.1 Corresponding the relationship between the content of CRF in arterial blood and histamine‐induced scratching number in mice at different time periods after acute stress

**FIGURE 1:**
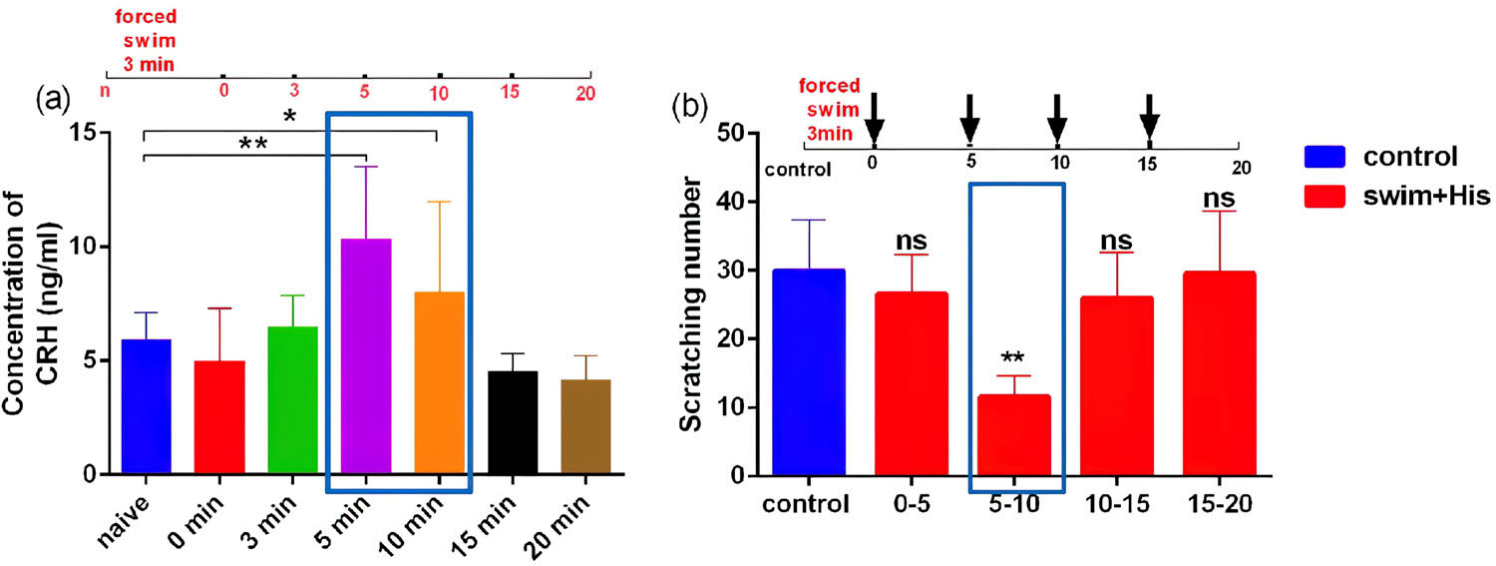
Changes of serum CRF content at different time points in mice after forced swimming and the number of scratches caused by intradermal injection of histamine (*n* = 6). (a) Serum CRF content at different time points after forced swimming (black box shows the time period when the CRF content is significantly increased); (b) the number of scratches caused by intradermal injection of histamine in mice after forced swimming (the black box shows the time period when the number of scratches was significantly inhibited). Black arrows indicate the time points after histamine injection. *P < .05 versus control; **P < .01 versus control. *Abbreviation*: ns, no significance versus control.

Three‐minute forced swimming (which does not form negative emotions and affect exercise capacity) was performed to simulate strong acute stress. We first observed the change in serum CRF content in mice undergoing forced swimming for 3 min. ELISA results showed that the content of CRF in ocular arterial blood of mice reached the peak at 5 min after forced swimming (*P* < .01, Figure 1a), decreased slightly at 10 min (*P* < .01, Figure 1a). Compared with the control group, there was no significant difference in CRF content at other time points (*P* > .05, Figure 1a). The scratching behavior results showed that the number of scratches caused by intradermal injection of histamine in mice was significantly reduced compared with the control group in the 5–10 min period after forced swimming (*P* < .01, Figure 1b). In other time periods (0–5 min, 10–15 min, and 15–20 min), there was no significant difference in the number of scratches between mice and controls (*P* > .05, Figure 1b). It is suggested that there is a corresponding relationship between the content of CRF in the blood of mice and the scratching behavior caused by histamine: 5–10 min after acute stress, with the sharp increase of CRF content, the acute itch caused by histamine is significantly inhibited; in other time periods, the content of CRF did not change significantly during the time period, nor did histamine‐induced scratching behavior.

### 3.2 Effects of acute stress on pain threshold in mice

**FIGURE 2:**
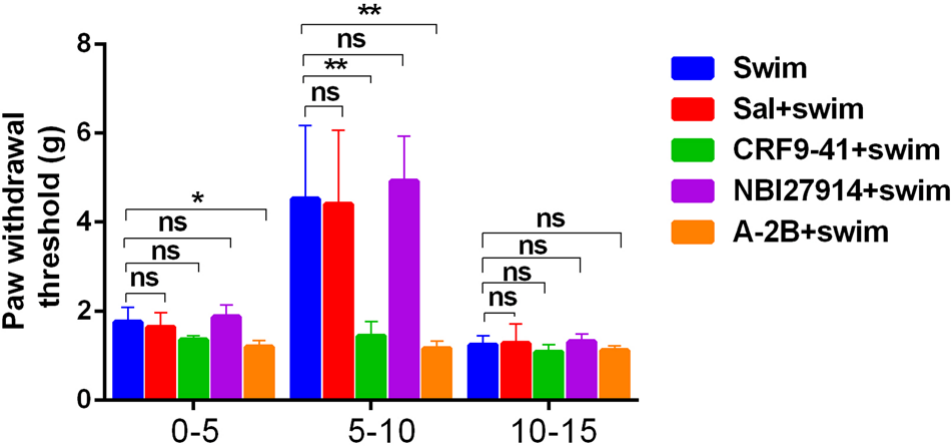
Mechanical pain threshold in different time periods after forced swimming in mice and the effect of intrathecal injection of CRF receptor antagonist on pain threshold (*n* = 6). The black frame shows that the pain threshold of mice was significantly increased in the 5–10‐min time period after forced swimming, and the pain threshold was significantly reduced after intrathecal injection of CRF9‐41 or A‐2B, but no effect of intrathecal injection of normal saline or NBI27914. **P < .01 versus control. *Abbreviation*: A‐2B, CRFR2‐specific antagonist; CRF9‐41, nonselective CRF receptor antagonist; NBI27914, CRFR1‐specific antagonist; ns, no significance.

According to the changes of serum CRF content of mice with acute stress in different periods, we further observed the change in mechanical pain threshold of mice in the swim group at different time periods after forced swimming (as shown in the three blue histograms in Figure 2). The results showed that the mechanical pain threshold of mice in the 5–10‐min time period after forced swimming was significantly higher than that in the 0–5 min and 10–15‐minutes time periods (*P* < .01). In the 5–10‐min time period after forced swimming, the mice in the CRF9‐41 + swim group (green histograms in Figure 2) and A‐2B + swim group (orange histograms in Figure 2) exhibited significant lower mechanical pain threshold compared those in the sal + swim group, swim group, and NBI27914 + swim group (*P* < .01). There was no significant difference in mechanical pain threshold among mice in the sal + swim group, swim group, and NBI27914 + swim group. There was no significant difference in pain thresholds among groups in the 0–5 min and 10–15 min time periods (*P* < .01, Figure 2). Given above, pain sensation is significantly inhibited 5–10 min after acute stress, which may be related to the sharp increase of serum CRF content which exerts pain modulation via CRFR2.

### 3.3 Effects of acute stress on itch sensation in mice

**FIGURE 3:**
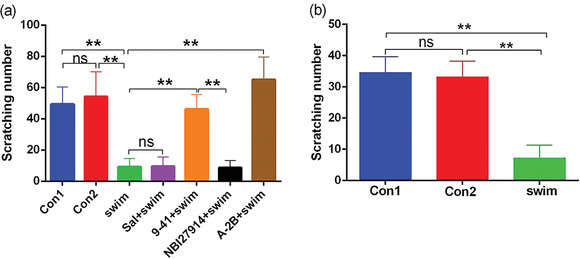
Effects of forced swimming on acute pruritus induced by histamine or chloroquine (*n* = 6). (a) The mice in each group were injected with histamine 5 min after the end of different treatments, and the number of scratches within 10 min after injection was recorded; (b) The mice in each group were injected with chloroquine 5 min after the end of different treatments, and the number of scratches within 10 min after injection was recorded. **P < .01. *Abbreviations*: A‐2B+swim, intrathecal injection of A‐2B group after forced swimming; Con 1, normal mouse group; Con 2, water‐sprayed simulated wet‐haired mouse group; swim, forced swimming group; ns, no significance; Sal + swim, intrathecal saline injection control group after forced swimming; 9–41+swim, intrathecal injection of 9–41 after forced swimming; NBI27914+swim, intrathecal injection of NBI27914 group after forced swimming.

According to the changes of serum CRF content in mice undergoing acute stress at different time periods, we selected the time point 5 min after the end of various treatments including forced swimming and performed intradermal injection of histamine or chloroquine in mice of different treatment groups, and the number of scratches within 10 min was counted. Behavioral results showed that compared with the normal control group (Con1) and the wet hair control group (Con2) (no significant difference seen between the two groups), mice in the acute stress group (swim) mice exhibited significantly decreased number of scratches was (*P* < .01) when treated with histamine (Figure 3a) or chloroquine (Figure 3b). Intrathecal injection of CRF9‐41 or A‐2B could reverse the inhibitory effect of acute stress (forced swimming) on histamine itch (*P* < .01, Figure 3a), and the reverse effect of A‐2B was higher than that of CRF9‐41 (*P* < .05). No difference in the degree of inhibition of histamine itch (*P* > .05, Figure 3a) was seen among the mice in the acute stress group (swim group), the saline intrathecal injection group (Sal + swim group), and the NBI27914 intrathecal injection group. Therefore, acute stress may also inhibit itch sensation. The inhibitory effect occurs in the 5–10‐min period after forced swimming, which may also be related to the sharp increase of serum CRF content and subsequent CRFR2‐mediated signaling.

### 3.4 Effects of intraventricular injection of CRF on acute itch induced by histamine or chloroquine

**FIGURE 4:**
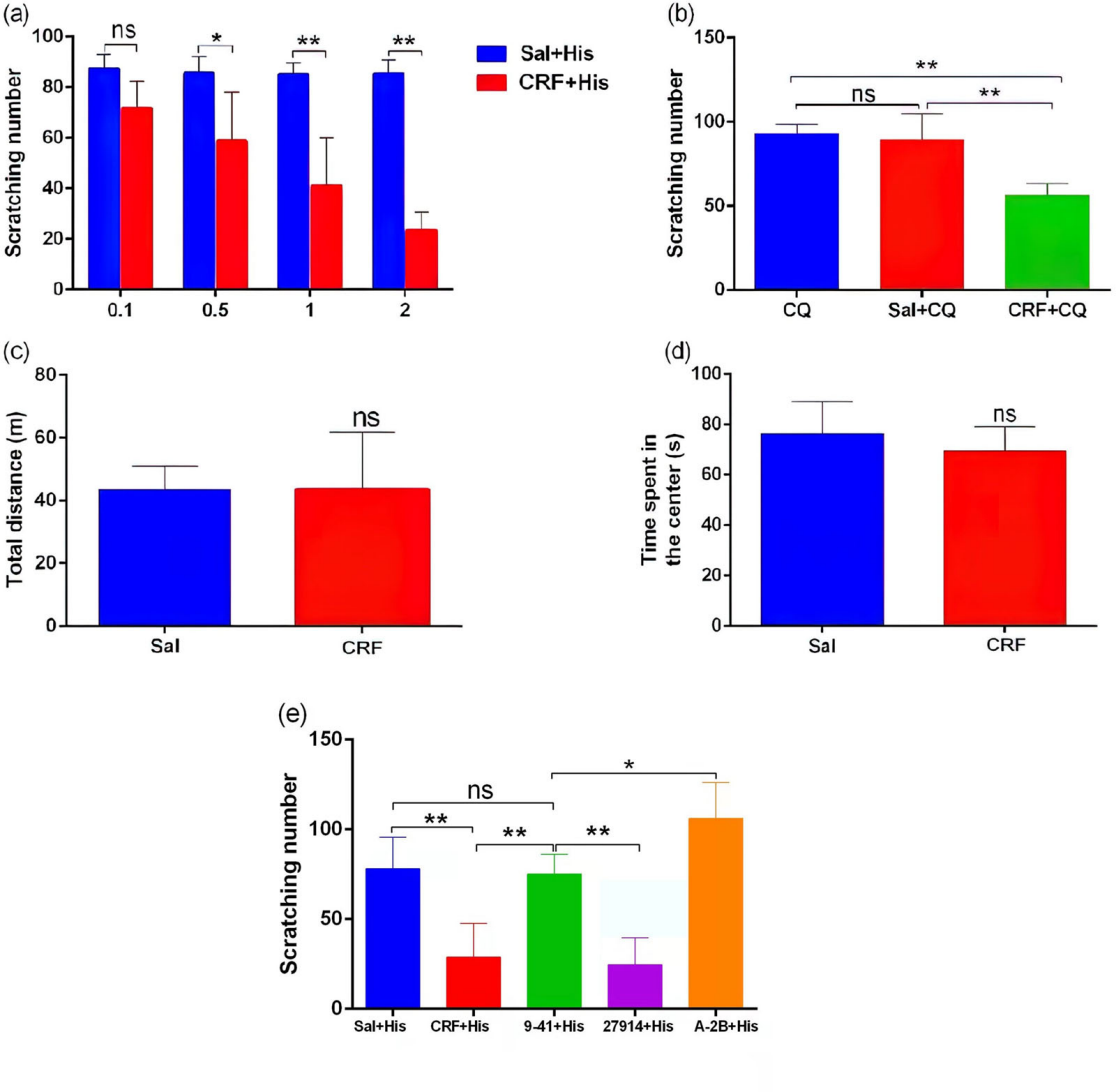
Effects of intraventricular injection of CRF on acute itch induced by histamine or chloroquine (*n* = 5). (a) The effect of intraventricular injection of different doses of CRF or normal saline on histamine itch; (b) the effect of intraventricular injection of 2 µl CRF (0.1 nmol/µl) or normal saline on acute pruritus induced by chloroquine; (c–d) comparison of total distance (c) and the activity time in the central area of the open field (d) after intraventricular injection of CRF or saline in the open field experiment; (e) intracerebroventricular injection of CRF9‐41, NBI27914, and A‐2B reversed the inhibitory effects of CRF on histamine‐induced pruritus. *P < .05; **P < .01. *Abbreviation*: ns, no significance.

In order to simulate the effect of massive release of CRF in the central nervous system during acute stress, we injected CRF into the ventricle to observe its effect on itch perception. We injected different doses of CRF into the ventricle of mice and observed its effect on acute itch induced by histamine or chloroquine. The results showed that the inhibitory effect of CRF on histamine itch was dose dependent, and the inhibitory effect began to appear when 0.5 µl CRF (0.1 nmol/µl) was injected into the ventricle, and the inhibitory effect on histamine itch gradually increased with the increase of the dose. The inhibitory effect was the strongest when the dose reached 2 µl (*P* < .05 or .01, Figure 4a), while the intraventricular injection of different doses of normal saline had no inhibitory effect on histamine itch (blue columns in the figure, Figure 4a). Intracerebroventricular injection of 2 µl CRF could also significantly reduce the number of scratches caused by chloroquine (*P* < .01, Figure 4b), while the intraventricular injection of an equal volume of normal saline could not inhibit chloroquine‐induced pruritus (*P* > .05, Figure 4b). In addition, when low doses (< 0.1 µl) of CRF were injected, no itch‐promoting effect was observed (results not shown).

At the same time, we detected no significant difference in the locomotion (total traveling distance) and exploratory behaviors (time spent in the central area) of the mice after intraventricular injection of CRF compared with saline injection (*P* > .05, Figure 4c,d). These suggest CRF injection at proper concentration do not cause changes in motor ability and anxiety in mice.

To demonstrate that the inhibitory effect of intraventricular injection of CRF on itch was mediated through CRF receptors, we injected the CRFR nonspecific antagonist CRF9‐41, the CRFR2‐specific antagonist A‐2B, and the CRFR1‐specific antagonist NBI27914 into the ventricle, respectively, and then observe the effect of CRF on histamine itch. The results showed that intraventricular injection of CRF9‐41 and A‐2B, instead of NBI27914, could completely reverse the inhibitory effect of CRF on histamine itch, and the inversion effect of A‐2B was better than that of CRF9‐41 (*P* < .01, Figure 4e). *P* < .05). These data suggested that intraventricular injection of higher concentrations of CRF can significantly inhibit the scratching response induced by pruritogenic agents, which is mediated by CRFR2.

## 4 DISCUSSION

CRF is widely distributed in the central nervous system. In the cerebral cortex, CRF‐positive neurons are scattered in layers I–VI. These neurons are relatively more abundant in layers II and III than layers IV and V, and very few such neurons could be found in layer VI (Merchenthaler, 1984). In subcortical brain regions, CRF is widely distributed in the paraventricular nucleus of the hypothalamus, central amygdala, hippocampus, bed nucleus of strial terminalis, locus coeruleus, spinal cord, DRG, and so forth. This distribution pattern determines functional diversity and complexity of CRF (Cummings et al., 1983; Smith & Vale, 2006). CRF is a hormone secreted by the paraventricular nucleus of the hypothalamus after external stress and participates in the stress response. In severe acute stress states (e.g., panic and nervousness), people often experience no pain or itch. For example, under the condition of war, the severe pain caused by mild trauma or even severe limb injury may not be felt in a short period of time. Strong acute stress leads to a sharp increase in CRF secretion (Nishioka et al., 1993), so whether this sharp increase in CRF is related to the decrease or even loss of somatosensory sensitivity such as pain and itch is worthy of systematic study.

Previous studies have observed that CRF is involved in the regulation of pain perception. For example, intraventricular injection of CRF can effectively inhibit pain behaviors caused by thermal experimental pain and inflammatory pain (Lariviere & Melzack, 2000). High doses of CRF, mimicking the condition of acute stress, results in increased pain thresholds in animals via acting on CRFR2 in the central amygdala (CeA), whereas low doses of CRF which is similar to the sustained release of chronic stress can lead to increased sensitivity to pain in rats via CRFR1 in CeA. This may explain that patients with depression, which results from long‐term exposure to chronic stress, often experience spontaneous pain in the absence of organic damage (Ji & Neugebauer, 2008).

At present, there is no research concerning the involvement of CRF in the regulation of itch under acute stress. In addition, very few studies centers on the involvement of CRF in the regulation of itch under chronic stress. It has been observed that after four weeks of water avoidance stress (WAS), the Nc/Nga mice (a model of congenital atopic dermatitis) spontaneously developed atopic dermatitis (AD) even in a specific pathogen‐free (SPF) environment, and this AD induced by WAS can be completely blocked by pre‐treatment with CRF; whereas normal Nc/Nga mice did not develop AD under SPF‐grade feeding conditions (Amano et al., 2008). Another study reported that persistently elevated CRF in patients with depression can directly act on the mast cells of the skin to cause their degranulation and induce pruritus (Theoharides et al., 1998).

In this study, a short‐term forced swimming (which does not form negative emotions and affect exercise capacity) was performed to simulate strong acute stress. Serum CRF content at different time points was detected by ELISA. We observed that the serum CRF content peaked 5–10 min after stress. During this period, acute itch caused by histamine or chloroquine, and mechanical pain caused by von Frey's stimulation were almost completely suppressed, which explains why high‐intensity acute stress can suppresses or even eliminates pain and itch sensations.

The CRF receptor (CRFR) includes three subtypes: CRFR1, CRFR2, and CRFR3. Among them, the distribution and function of CRFR3 are unclear. In order to verify which receptor mediates the analgesic and antipruritic effects, we injected selective and nonselective antagonists of CRFRs intrathecally and observed that either CRF9‐41 or A‐2B could abolish the effect of acute stress on acute pain, suggesting that analgesia and antipruritus exerted by acute stress are related with CRF. Further studies indicate these effects are mediated by CRFR2. Previous studies have shown that CRF exerted analgesic effect via CRFR2 and intraventricular injection of CRF could also exert analgesic effect. Thus, in this study we focused on the regulation effect of intraventricular injection of CRF (simulating acute stress condition) on itch sensation. We injected CRF into the ventricle of mice to simulate the excessive endogenous release of CRF and observed that the dose‐dependent inhibitory effect of CRF on histamine itch was mediated by CRFR2, which started at the dose of 0.5 µl CRF (0.1 nmol/µl) and reached the plateau at the dose of 2 µl, which was also mediated by CRFR2. Similarly, intracerebroventricular injection of 2 µl of CRF also significantly reduced the number of scratches induced by chloroquine. At the same time, CRF injection did not cause changes in motor ability and anxiety in mice, so as to exclude that the inhibition of pain and itch sensation by CRF was caused by motor dysfunction and depression in mice.

In conclusion, we conclude that both a sharp endogenous CRF release under the condition of acute stress and high exogenous CRF injection can inhibit itch and pain sensations, and this effect is mediated by acting on the CRFR2. As a hormone that widely exists in the human body and participates in various stress responses, the prospects of the clinical application of CRF cannot be ignored. This study mainly explored the role of CRF in stress analgesia and pruritus suppression through CRFR2, but downstream mechanisms of its analgesic and antipruritus effects need to be further explored. Other studies have confirmed that CRF is involved in the formation and regulation of depression, in which CRFR1 mainly plays a pro‐depressant role and CRFR2 mainly plays an antidepressant role (Fortune et al., 2003; Heijmans, 1998). This study is expected to provide theoretical and experimental basis for the treatment of stress, especially for those with stress disorders such as post‐stress depression and the accompanying pain or itch paresthesia.

## AUTHOR CONTRIBUTIONS

Xiao‐Dong Wang, Shang‐Qing Liu, and Hui Li were involved in the design of the study. Xiao‐Dong Wang, Hao‐Miao Bai, Xiao‐Lan Li, Lin‐Fang Zhang, and Zhen‐Yu Wu were involved in the behavior tests. Yang Bai and Fei Li were involved in the ELISA test. The draft manuscript was written, reviewed, and edited by Xiao‐Dong Wang, Hao‐Miao Bai, Shang‐Qing Liu, and Hui Li. All authors contributed and approved the final version of the manuscript.

## CONFLICT OF INTEREST

The authors declare that they have no conflict of interest.

### PEER REVIEW

The peer review history for this article is available at: https://publons.com/publon/10.1002/brb3.2783.

## Data Availability

I confirm that my article contains a Data Availability Statement even if no data is available (list of sample statements) unless my article type does not require one. cd_value_code=text. The data that support the findings of this study are available from the corresponding author upon reasonable request.
